# A Randomized Controlled Trial Evaluating the Effects of Diosmin in the Treatment of Radicular Pain

**DOI:** 10.1155/2017/6875968

**Published:** 2017-10-08

**Authors:** Yinhe Wang, Xin Fang, Lei Ye, Yishan Li, Hongfei Shi, Yang Cao

**Affiliations:** ^1^Department of Orthopedic Surgery, Nanjing Drum Tower Hospital, Nanjing University Medical School, Nanjing, Jiangsu 210008, China; ^2^Unit of Biostatistics, Institute of Environmental Medicine, Karolinska Institutet, 17177 Stockholm, Sweden; ^3^Department of Pharmacology, Qianfo Hill Institute of Shandong Province, Jinan, Shandong 250014, China; ^4^Department of International Training, PLA University of Science and Technology, Nanjing, Jiangsu 210007, China; ^5^Clinical Epidemiology and Biostatistics, School of Medical Sciences, Örebro University, 70182 Örebro, Sweden

## Abstract

Diosmin has been widely used to treat patients with vascular pain for its potent anti-inflammatory and analgesic effects. To evaluate the therapeutic effects of Diosmin in the treatment of radicular pain, we conducted an investigator-initiated, randomized, active-controlled noninferiority trial between January 1, 2009, and December 1, 2010. Diosmin (50 mg/kg/day) was orally administered to treat the radicular pain in 150 patients for one month. Another 150 patients with the same symptom were given 20% 250 ml mannitol (1 g/kg/day) for 7 days and dexamethasone (10 mg/day) for 3 days intravenously guttae. Short-term relief and long-term relief were measured. Secondary outcomes include improvement in functional and psychological status, return to work, and reduction in anti-inflammatory analgesic drugs intake. Patients treated with oral Diosmin achieved reduction in radicular pain. The total satisfaction rate of Diosmin group was 84.7% [95% confidence interval (CI): 77.9%, 90.0%], and the complete satisfaction rate was 50.7% (95% CI: 42.4%, 58.9%). No statistically significant difference was found between the Diosmin group and the active-control group regarding patient satisfaction. No adverse effects were found during the study period. Our study suggests that clinical application of Diosmin with a dose of 50 mg/kg/day might reduce the radicular pain. This trial is registered with ISRCTN97157037.

## 1. Introduction

Radicular pain is a common chronic pain, which is commonly caused by disc herniation, although a space occupying lesion in the lumbosacral spine or spondylolisthesis can also result in such clinical condition. The main cause of lumbosacral radiculopathy is intervertebral disc herniation. Among patients with lumbosacral radiculopathy, about 10–15% had surgery eventually, but overall the majority recovers with conservative management. In general, nonsteroidal anti-inflammatory drugs (NSAIDs) and acetaminophen are commonly used to treat inflammatory pain in clinical practices [1].

Studies suggest that the mechanism of radicular pain is due to the inflammatory nerve roots [2]. Chemical radiculitis, a chemically mediated noncellular inflammatory reaction, which may occur through a disc rupture and imply irritation of the nerve root by perineural spread of nucleus pulposus, might be a complementary explanation. Nucleus pulposus is inflammatogenic and leukotactic [3]. Previous study shows that inflammatory processes participate in nerve pain to secrete proinflammatory cytokines like 5-HT and TNF and suppress anti-inflammatory factors such as interleukin-10 [4]. Mechanical compression and hypoxia, directly or indirectly caused by disc herniation, blood supply, and cerebrospinal fluid flow of spinal nerve roots and peripheral nerves are related to abnormal sensations and pain, shown as possible causes of symptoms [5].

Drug therapy, physical therapy, and psychotherapy, surgery, bed rest, acupuncture, spinal cord stimulation, cryotherapy, and radiofrequency thermocoagulation have been used for treating radicular pain [6]. For more than 50% of patients with the symptom, physicians suggest using simple analgesics and resuming daily activities. Although there is only limited evidence of the long-term effectiveness, opioids analgesic has also been used to treat radicular pain. However, some negative outcomes, for example, higher risk for developing problematic opioid use or addiction during long-term opioid therapy, have been observed in patients with mental and substance use disorders [7].

Anti-inflammatory cytokine therapy may be an effective treatment of sciatica which resulted from disc herniation due to its capability of preventing the dorsal root ganglion compartment syndrome, which might be a side effect of applying nucleus pulpous topically. Flavonoids, a group of plant extracts, have been widely used in biochemistry and pharmacology because of their anti-inflammatory, immunomodulatory, and antioxidant effects in vivo and in vitro [8]. Flavonoids may improve lymphatic drainage by significantly increasing lymphatic flow. Their purified micronized compound has been used to treat chronic venous insufficiency and the beneficial effects have implication in treating disc herniation related sciatica.

Although Diosmin has been widely used in treating various pains for patients with intravenous vascular pain, to the best of our knowledge, there is no published data so far for the indication of radicular pain. We compared the degree of pain relief and physical function improvement in the radicular pain patients treated by Diosmin and an active treatment in the presented study.

## 2. Materials and Methods

### 2.1. Study Design

This investigator-initiated, randomized, active-controlled, noninferiority trial was carried out in the Department of Orthopedic Surgery, Drum Tower Hospital of Nanjing University Medical School between January 1, 2009, and December 1, 2010. The protocol was approved by the Ethics Committees of Drum Tower Hospital of Nanjing University Medical School. Three hundred consecutive outpatients with radicular pain (previously diagnosed with unilateral leg pain greater than low back pain, positive straight leg raising test, radiating pain in lower limbs, numbness, and paresthesia in the same distribution) [9] and/or evidence of nerve root compromise, lumbar disc degeneration, herniation, or protrusion on magnetic resonance scan were enrolled. Informed signed consent was obtained from all the patients. The trial was registered at the ISRCTN registry (number ISRCTN97157037) “(http://www.isrctn.com/ISRCTN97157037)”.

### 2.2. Inclusion and Exclusion Criteria

The inclusion criteria were preliminarily diagnosis of radicular pain; age ≥ 18 years; pain duration ≥ 30 days; being previously treated with nonsteroidal anti-inflammatory drugs, opioid medications, or physical therapy without pain relief for more than one month; being with normal or slight decrease in the height of disc space on lateral plain X-ray film; and being initially considered eligible for surgical intervention.

Based on patient's history and clinical and imaging examinations, the exclusion criteria were patients with spondylolysis, lumbar canal stenosis, isthmic or degenerative spondylolisthesis, inflammatory arthritis, spinal instability, infection, previous lumbar surgery, neurologic disease, tumor, or psychological disorders (such as depression or using antidepressants or anxiolytic medications). Patients with lumbar intervertebral disc protrusion who were diagnosed according to magnetic resonance by a radiologist and an orthopedic surgeon and needed surgery were also excluded.

### 2.3. Treatments

Three hundred outpatients with radicular pain were randomly assigned into the Diosmin group and the active-control, that is, mannitol and dexamethasone [10], group with 1 : 1 ratio using computer-aided random assignment. Diosmin group received Diosmin (Nanjing Chia Tai Tianqing Pharmaceutical Co., Ltd.) per os, 900 mg, tid for 2 weeks followed by bid for 2 weeks, and lastly 450 mg bid as maintenance dose for at least one month [11, 12]. Control group received 20% mannitol (CR Double-Crane Pharmaceuticals Co., Ltd.) 250 ml (1 g/kg/day) and dexamethasone (Furuitang Pharmaceutical Co., Ltd.) 10 mg/day intravenously guttae for the first 3 days and followed by mannitol for 4 days. In both groups, the courses of treatment lasted at least one month. The patients with serious pain [visual analog scale (VAS) > 8 in our study] were given diclofenac (Novartis) 75 mg/day for maximum 7 days. In case symptoms appeared again one month after last symptoms disappeared, the same regimen was given again.

### 2.4. Outcomes and Follow-Ups

*Short-Term Outcomes*. A visual analog scale (VAS) was used to measure the main outcomes [13]. To measure the pain that the patient had ever perceived during a particular time period, a standard 10-centimeter line was used in the VAS in which 0–0.4 cm meant no pain and 7.5–10 cm meant severe pain. A numerical rating scale (NRS) [13], with points ranging from 0 (no pain) to 100 (worst pain experienced in the last 1 week), was used. VAS and NRS outcome measures were collected just after the procedure, weeks 2 and 8 after procedure, respectively. To comply with the conventional scaling, VAS scores were rescaled to 0–100 and NRS scores to 0–10. The Roland-Morris Questionnaire (RM-Q) [14] was filled by the patient thrice, just after procedure and weeks 2 and 8 too.

*Long-Term Outcomes*. The patients had regular visit to the outpatient department of the hospital or we took regular telephone follow-up for those who did not have regular visit. Current pain level and satisfaction were recorded during each visit or follow-up for every patient. The long-term outcomes were evaluated in the 24th month after randomization by hospital visit or telephone interview. Medication usage and posttreatment satisfaction were examined. Usage of NSAIDs or opioid medications was graded as follows: 1, never; 2, occasional; 3, regular. A three-grade scale was used to rate patient's satisfaction: 1, completely satisfied (no pain at all time and no restriction of activities); 2, satisfied (slight pain that requires no medication and mild restriction of activities); 3, unsatisfied (moderate to severe pain that requires medication and moderate to severe restriction of activities) [15].

*Adverse Effect*. Adverse effects, including gastrointestinal discomfort, were recorded in adverse effects form through patients' complaint during outpatient department visit or follow-up.

To minimize investigator bias, the independent investigator who recorded all the results was blinded to both therapeutic groups.

### 2.5. Statistical Analysis

Intention-to-treat analysis was used to avoid various misleading artifacts that could arise in the trial such as nonrandom attrition of the patients. Missing values were imputed by the last-observation-carried-forward method as recommended [16]. All results were expressed as mean ± standard deviation (SD) for continuous variables and proportion for categorical variables. Student's *t*-test and *χ*^2^ test were used for statistical comparisons for continuous variables and categorical variables, respectively. VAS, NRS, and RM-Q scores of pretreatment and posttreatment at different time points were presented as means with corresponding 95% confidence intervals (CIs) and compared using two-way analysis of variance. Two-sided *P* values < 0.05 and one-sided *P* values < 0.025 were considered statistically significant for baseline comparison and for posttreatment comparison, respectively. Statistical software programmes IBM SPSS 24 (IBM Corporation, Armonk, New York) and Stata 14.2 (StataCorp LLC, College Station, Texas, USA) were used for all the analyses.

## 3. Results

### 3.1. Characteristics of Patients at Baseline

In total, 300 patients with radicular pain were included in the study and were randomly assigned into the Diosmin group (*n* = 150) and the active-control group (*n* = 150). The baseline characteristics were balanced between the two groups and no statistically significant difference was found (Table 1). The age of patient is 41 ± 6.5 years and 42 ± 7.4 years (*t* = 1.244, *P* = 0.215) in the Diosmin group and the active-control group, respectively. The courses of treatment are 4 ± 1.4 months and 4 ± 1.5 months (*t* = 0.000, *P* = 1.000) in the Diosmin and the active-control group, respectively. Duration of pain ranged from 1 month to 48 months and there was no statistically significant difference between two groups (*χ*^2^ = 0.546, *P* = 0.909). The level of lesion and baselines pain score for both groups are shown in Table 1. Statistically significant difference was only found for VAS (*t* = 2.093, *P* = 0.037; Table 1).

### 3.2. Clinical Outcome

All the patients completed three follow-up visits as planned and there was no drop out during the study period. In both groups, there was statistically significant improvement in the RM-Q, NRS, and VAS scores at week 2 and week 8 of posttreatment compared to pretreatment (Table 2). Improvement in RM-Q, NRS, and VAS scores retained during follow-up and was statistically significant (Table 2).

The difference of patient satisfaction and other medication usage between the two groups at the 24 months after randomization were not statistically significant (Table 3).

Frequency of overall medication usage (narcotics, or opioid medications, or both opioid medications and NSAIDs) after treatment decreased notably in both groups (data not shown). Again, no statistically significant difference was found between the two groups (Table 4).

### 3.3. Adverse Effects

There was no serious adverse effect in both groups. Five patients in the control group reported mild nausea without vomiting. The symptoms disappeared after drug withdrawal and no special treatment was required.

## 4. Discussion

A significant proportion of patients suffering from chronic pain with radicular component have a high disability index [17, 18]. Studies have indicated that potential causes of radicular pain include nerve root compression, reduced blood flow, increased endoneurial edema, and fluid pressure in associated nerve roots, which may cause neuronal ischemia and/or sensory disorder [19]. The treatment of radicular pain has traditionally been limited to either conservative management or surgical methods [20]. In general, patients with radicular pain can be treated conservatively. In recent decades, invasive procedures such as lumbar spine surgery have been performed increasingly on patients with radicular pain; however radicular pain was observed after operations [21]. Radicular pain may sometimes become more severe following a steroid epidural block. Side effects after using steroids have been related to the chemistry and pharmacology of the steroids [22]. A recent 1-year follow-up study found a recovery rate of 95% among patients with sciatica randomized to either early surgery or prolonged conservative treatment [23]. Another report found that patients with lumbar intervertebral disc herniation improved substantially after either surgical or conservative treatment [24]. However, compared to the patients treated conservatively, work or disability outcomes at 2 years either with or without work compensation were not improved for patients treated surgically [24].

Reports showed that conservative treatment for radicular pain had satisfactory results. Besides treating medical and psychiatric comorbidities, conservative treatments include physiotherapy, meditation, transcutaneous electrical nerve stimulation, and relaxation techniques, activity, exercise, and/or stretching to reduce spinal extension [25]. Although oral analgesics (usually NSAIDs and opioids) are prescribed frequently, the effectiveness is limited. Both analgesics might have substantial side effects after long-term usage. Regarding whether the benefit of one class is over another, there is no conclusive evidence. Studies have associated NSAIDs with gastrointestinal bleeding, renal impairment, and potentially elevated cardiovascular risk in the case of cyclooxygenase-2 inhibitors [26]. On the other hand, opioids have been associated with potential abuse and tolerance and dose-related risk of death [27]. Especially in the setting of long-term opioid therapy, some patient characteristics including mental disorders and substance abuse have been identified as risk factors associating with negative outcomes of problematic use or addiction in observational studies [7].

From chemical aspect, experimental studies have shown the spontaneous resorption of disk herniation [28] and the immunogenicity of intervertebral disk [29–32]. Inflammatory mediators including phospholipase A2, prostaglandin E2, interleukin- (IL-) 1a, IL-1b, IL-6, tumor necrosis factor- (TNF-) *α*, and nitric oxide (NO) have also been identified around and within intervertebral disk tissue. The evidence indicates that inflammatory factors TNF-*α* as well as NO, prostaglandin E2 (PGE2), and IL-6 are the main candidates among substances potentially responsible for nerve root pain according to the current pathophysiology [33–38]. The proinflammatory substances secreted by nucleus pulposus may also contribute to the damaged nerve roots in the absence of mechanical compression [39].

Our study indicates that patients treated orally with Diosmin achieved reduction in radicular pain, as measured by the NRS, RM-Q, and VAS. The satisfaction rates of the patients were 84.7% (95% CI: 77.9%, 90.0%) and 84.0% (95% CI: 77.1%, 89.5%) after 24 months of randomization for the Diosmin group and the active-control group, respectively. In general, all primary outcomes were similar between the Diosmin group and the control group without statistical significance. No serious adverse effects were found in both groups. However five patients in the control group reported mild nausea and the symptoms get disappeared after drug withdrawal and no special intervention was required.

Improved venous tone and capillary bed microcirculation, reduced capillary permeability, increased lymphatic drainage, and inhibited inflammatory reactions have been identified as main mechanisms of Diosmin's action [40]. Being potent inhibitors of PGE2 and thromboxane A2, Diosmin and other flavonoids may inhibit leukocyte activation, migration, and adhesion. Diosmin provides protection against microcirculatory damage through decreasing the plasma levels of endothelial adhesion molecules and reducing neutrophil activation significantly [41].

Although our study provided evidence that Diosmin may have similar pain relief effect compared with the active-control treatment mannitol plus dexamethasone, we have to acknowledge that our study was a single center trial and limited by the small number of patients. Further multicenter randomized controlled clinical trial with larger sample size is required to confirm the clinical application of Diosmin in treating radicular pain.

## 5. Conclusions

As a potential promising therapeutic drug, Diosmin may reduce radicular pain that might partly be attributed to its anti-inflammatory and analgesic components. Its effects are similar to currently used active treatment mannitol plus dexamethasone.

## Figures and Tables

**Table 1:** Baseline characteristics of the patients.

	Diosmin group *n* = 150	Control group *n* = 150	*P* value
Male (*n*, %)	76, 50.7%	73, 48.7%	0.729
Age (years, mean ± SD)	41 ± 6.5	42 ± 7.4	0.215
Treatment course (months, mean ± SD)	4 ± 1.4	4 ± 1.5	1.000
Duration of pain (*n*, %)			
<3 months	45, 30.0%	47, 31.3%	0.909
3–12 months	62, 41.3%	65, 65%	
13–36 months	29, 19.3%	27, 18.0%	
>36 months	14, 9.3%	11, 7.3%	
Pretreatment with opioids (*n*, %)			
None	118, 78.5%	114, 76.0%	0.849
<60 morphine equivalents/day	21, 14.0%	23, 15.3%	
≥60 morphine equivalents/day	11, 7.3%	13, 8.7%	
Current smoker (*n*, %)	21, 14.0%	23, 15.3%	0.744
Obesity (*n*, %)	23, 15.3%	25, 16.7%	0.753
Level of lesion (culprit nerve root, *n*, %)			
L2-3/L3	9, 6.0%	11, 7.3%	0.788
L3-4/L4	13, 8.7%	15, 10.0%	
L4-5/L5	99, 66.0%	101, 67.3%	
L5-S1/S1	29, 19.3%	23, 15.3%	
Baseline pain scores (mean ± SD)			
VAS	89 ± 11	91 ± 4	0.037
RM-Q	17.2 ± 2.7	17.4 ± 2.3	0.490
NRS	7.8 ± 0.7	7.8 ± 0.3	0.222
Number of patients with serious pain given diclofenac (*n*, %)	6, 3.9%	5, 3.3%	0.759

SD, standard deviation; VAS, visual analog scale; RM-Q, Roland-Morris Questionnaire; NRS, numerical rating scale.

**Table 2:** Comparisons of mean RM-Q, NRS, and VAS scores (95% CI) between pre- and posttreatment.

Outcome	Diosmin			Control		
	Pretreatment	2 weeks	8 weeks	Pretreatment	2 weeks	8 weeks
RM-Q	17.2 (16.8, 17.6)	11.8 (11.3, 12.3)^*∗*^	9.1 (8.7, 9.5)^*∗*^	17.4 (17.0, 17.8)	11.3 (10.9, 11.7)^*∗*^	8.1 (7.6, 8.6)^*∗*^
NRS	7.8 (7.7, 7.9)	5.7 (5.5, 5.8)^*∗*^	3.4 (3.3, 3.5)^*∗*^	7.8 (7.7, 7.9)	5.4 (5.3, 5.4)^*∗*^	3.2 (3.1, 3.3)^*∗*^
VAS	89 (87, 91)	52 (51, 53)^*∗*^	36 (34, 38)^*∗*^	91 (90, 92)	55 (53, 57)^*∗*^	34 (33, 35)^*∗*^

^*∗*^One-sided *P* < 0.025, within group comparison was performed using two-way analysis of variance. RM-Q, Roland-Morris Questionnaire; NRS, numerical rating scale; VAS, visual analog scale.

**Table 3:** Patient satisfaction and other medication usage at the 24th month after randomization.

	Diosmin	Control	*P* value
Satisfaction of patients: *n*, % (95% CI)			
Completely satisfied	76, 50.7% (42.4%, 58.9%)	77, 51.3% (43.0%, 59.6%)	0.967
Satisfied	51, 34.0% (26.5%, 42.2%)	49, 32.7% (25.2%, 40.8%)	
Unsatisfied	23, 15.3% (10.0%, 22.2%)	24, 16.0% (10.5%, 22.9%)	
Other medication usage: *n*, % (95% CI)	46, 30.7% (23.4%, 38.7%)	43, 28.7% (21.6%, 36.6%)	0.705
Nonsteroidal anti-inflammatory drugs: *n*, % (95% CI)	41, 27.3% (20.4%, 35.2%)	39, 72.7% (19.2%, 33.8%)	0.794
Opioid medications: *n*, % (95% CI)	33, 22.0% (15.7%, 29.5%)	36, 24.0% (17.4%, 31.6%)	0.681

**Table 4:** Frequency of overall medication usage at the 24th month after randomization: *n*, % (95% CI).

Overall medication usage	Diosmin	Control	*P* value
None	104, 69.3% (61.3%, 76.6%)	107, 71.3% (63.4%, 78.4%)	0.845
Occasional	33, 22.0% (15.7%, 29.5%)	29, 19.3% (13.3%, 26.6%)	
Regular	13, 8.7% (4.7%, 14.4%)	14, 9.3% (5.2%, 15.2%)	

## Abbreviations

NSAID: Nonsteroidal anti-inflammatory drug
VAS: Visual analog scale
NRS: Numerical rating scale
RM-Q: Roland-Morris Questionnaire
SD: Standard deviation
IL: Interleukin
TNF: Tumor necrosis factor
NO: Nitric oxide
PGE2: Prostaglandin E2.
